# A nomogram for predicting prognosis of multiple myeloma patients based on a ubiquitin-proteasome gene signature

**DOI:** 10.18632/aging.204432

**Published:** 2022-12-18

**Authors:** Dexiang Ji, Yong Liu, Wenjie Sun, Qing Shi, Guoan Chen, Zhiwang Song, Yanxia Jiang

**Affiliations:** 1 Department of Hematology, First Affiliated Hospital of Nanchang University, Nanchang 330006, Jiangxi, China; 2 Department of Emergency, First Affiliated Hospital of Nanchang University, Nanchang 330006, Jiangxi, China; 3 Department of Anesthesiology, First Affiliated Hospital of Nanchang University, Nanchang 330006, Jiangxi, China; 4 Department of Endocrinology and Metabolism, First Affiliated Hospital of Nanchang University, Nanchang 330006, Jiangxi, China; 5 Department of Oncology, First Affiliated Hospital of Nanchang University, Nanchang 330006, Jiangxi, China

**Keywords:** multiple myeloma, ubiquitin-proteasome system, prognosis, visualization, signature

## Abstract

Background: Multiple myeloma (MM) is a malignant hematopoietic disease that is usually incurable. However, the ubiquitin-proteasome system (UPS) genes have not yet been established as a prognostic predictor for MM, despite their potential applications in other cancers.

Methods: RNA sequencing data and corresponding clinical information were acquired from Multiple Myeloma Research Foundation (MMRF)-COMMPASS and served as a training set (n=787). Validation of the prediction signature were conducted by the Gene Expression Omnibus (GEO) databases (n=1040). To develop a prognostic signature for overall survival (OS), least absolute shrinkage and selection operator regressions, along with Cox regressions, were used.

Results: A six-gene signature, including KCTD12, SIAH1, TRIM58, TRIM47, UBE2S, and UBE2T, was established. Kaplan-Meier survival analysis of the training and validation cohorts revealed that patients with high-risk conditions had a significantly worse prognosis than those with low-risk conditions. Furthermore, UPS-related signature is associated with a positive immune response. For predicting survival, a simple to use nomogram and the corresponding web-based calculator (https://jiangyanxiamm.shinyapps.io/MMprognosis/) were built based on the UPS signature and its clinical features. Analyses of calibration plots and decision curves showed clinical utility for both training and validation datasets.

Conclusions: As a result of these results, we established a genetic signature for MM based on UPS. This genetic signature could contribute to improving individualized survival prediction, thereby facilitating clinical decisions in patients with MM.

## INTRODUCTION

As a hematologic malignancy, Multiple myeloma (MM) accounts for 1.3% of all malignancies and 15% of hematologic neoplasms, with an incidence of 4.5 to 6 cases per 100,000 inhabitants [1]. As a malignant clonal plasma cell disease, MM originates in the bone marrow and the main clinical manifestations are the accumulation of clonal plasma cells predominantly in bone marrow, triggering the overproduction of nonfunctional intact immunoglobulins or immunoglobulin chains [2–4]. With improved understanding of MM and the application of new drugs and treatments, MM patient survival has increased in recent years [2]. Despite recent progress in treatment, MM remains incurable with high recurrence rates and drug resistance rates, with a median survival time of only 5-6 years [5], and the pathogenesis has not been elucidated [6]. Therefore, it is vital to research the complex biology and heterogeneous clinical course of MM, and research novel biomarker to better predict MM patients’ prognosis.

The ubiquitin proteolysis system (UPS) plays a crucial role in regulating targeted protein degradation in eukaryotes, thus is essential for maintenance of protein homeostasis at the level of protein degradation. The UPS consists of numerous proteins, including ubiquitin-activating enzymes (E1), ubiquitin-conjugating enzymes (E2), and ubiquitin ligases (E3) [7]. In the biochemical physiology, the enzymes E1 and E2 prepare ubiquitin for conjugation, while E3 is responsible for recognizing the specific substrate before catalyzing the transfer of activated ubiquitin to it [8]. Recently, there is accumulating evidence that ubiquitylation plays a crucial role in cancer pathogenesis and that targeting ubiquitylation may provide a very promising therapeutic approach in a variety of cancers [9–12]. For instance, the NF-κB pathway, which is frequently altered in MM, is highly regulated by ubiquitination. Thus, the UPS provides many opportunities for pharmacologic intervention. In the past two decades, proteasome inhibitors have emerged as one of the most important classes of agents for treating MM [13]. There are several downstream effects of inhibiting the proteasome, including the inhibition of NF-κB signaling, etc. UPS related genes are the key regulators of ubiquitin proteolysis system [14]. So far, the clinical significance of UPS genes has not been systematically investigated in patients with MM.

In the present study, we established and validated a UPS gene signature for predicting MM patient outcomes, and then built a nomogram by classifying patients based on UPS signature risk score and other clinicopathological factors to improve our ability to predict the survival of MM cases, and could guide comprehensive MM therapeutic strategies. The visualization model was created using a web-based calculator, and the estimation performance was assessed based on discrimination, calibration, and clinical value.

## MATERIALS AND METHODS

### Data collection

The transcriptome and clinical data, including survival information, are publicly available through the Multiple Myeloma Research Foundation (MMRF) CoMMpass data (https://research.themmrf.org) and Gene Expression Omnibus (GEO) database (https://www.ncbi.nlm.nih.gov/geo/). MMRF-COMMPASS dataset which contains 787 cases with MM was set as the training set. Three independent datasets, GSE2658 (n=559), GSE136377 (n=426), and GSE57317 (n=55) were set as the validation sets. GSE118985 included bone marrow samples from 68 normal controls and 460 newly diagnosed patients with MM and 132 MM patients in complete remission. A list of 804 UPS genes (Supplementary Table 1) were identified in the previous studies and used as the basis of our evaluations in the current study [15, 16].

### Construction prognostic UPS signature

To narrow the range of candidate prognostic UPS genes, we first performed univariate Cox analyses based on MMRF-COMMPASS and GSE2658 by the “survival” package. The overlapping prognostic genes in MMRF-COMMPASS and GSE2658 was selected for subsequent studies. By using the R packages “glmnet” and “survival,” the LASSO regression analysis was carried out to screen potential genes based on variable screening and complexity adjustment. Finally, we conducted multivariate Cox regression analyses to identify highly correlated genes and construct the UPS gene signature on the basis of the following equation for risk scores:


$$\text{Risk}\;\text{score}=\text{Risk}\;\text{score}=\sum_{\text{i}=0}^{N}\left(\beta_{\text{i}}\times E{\text{xp}}_{i}\right)$$

In this equation, βi denotes the regression coefficient, i denotes the UPS genes used to construct the signature, Exp denotes the relative expression value of each UPS gene in the signature, whereas N signifies the sum of genes within the signature. Patients were divided into two groups according to their median risk scores: low-risk and high-risk, and the “survivalROC” package in R was used to create the receiver operating characteristic (ROC) curves. To evaluate the predictive power of the UPS gene signature, AUC values were calculated.

### Gene set variation analysis (GSVA)

We use “GSVA” package calculate the concentration of each sample in the gene enrichment of scoring, and then predefined gene rank. Specifically, we first use of gene expression profile, Using Hanzelmann et al. method and obtained Molecular Signatures Database to download the c2. Cp. Kegg. V7.4. symbols.gmt and c6.all.v7.4.symbols.gmt collections [17], which were used to evaluate related pathways and molecular mechanisms. The minimum gene set was set as 5, and the maximum gene set was set as 5000. The enrichment score of each sample in each gene set was calculated.

### Construction and evaluation of the nomogram

We used univariate and multivariate Cox regression analyses to evaluate the independent prognostic value of the risk score. In the training set, a nomogram was developed based on independent risk factors, and risk scores were calculated for each patient [18, 19]. Training and validation sets were used to estimate the accuracy of the nomogram model. The web-based calculator for was built through package “DynNom”.

### Immune cell infiltration

The immune cell infiltration in the high- and low-risk groups was calculated by the Cell-type Identification by Estimating Relative Subsets of RNA Transcripts (https://cibersort.stanford.edu/) [20]. Differences in the relative proportions of 22 types of immune cells between the high- and low-risk groups was calculated based on the absolute mode between the low- and high-risk groups.

### Statistical analysis

Kaplan-Meier (KM) curves were plotted when median risk scores were used as cutoffs for comparing high-risk and low-risk survival rates. Statistics were considered significant for results with p values less than 0.05. R software version 4.2.1 is used for all analyses, with the exception of instructions performed with special parameters.

### Availability of data and materials

All the data were obtained from the MMRF-CoMMpass data (https://research.themmrf.org) and GEO database (https://www.ncbi.nlm.nih.gov/geo/).

## RESULTS

### Construction of UPS risk signature

**Figure 1:**
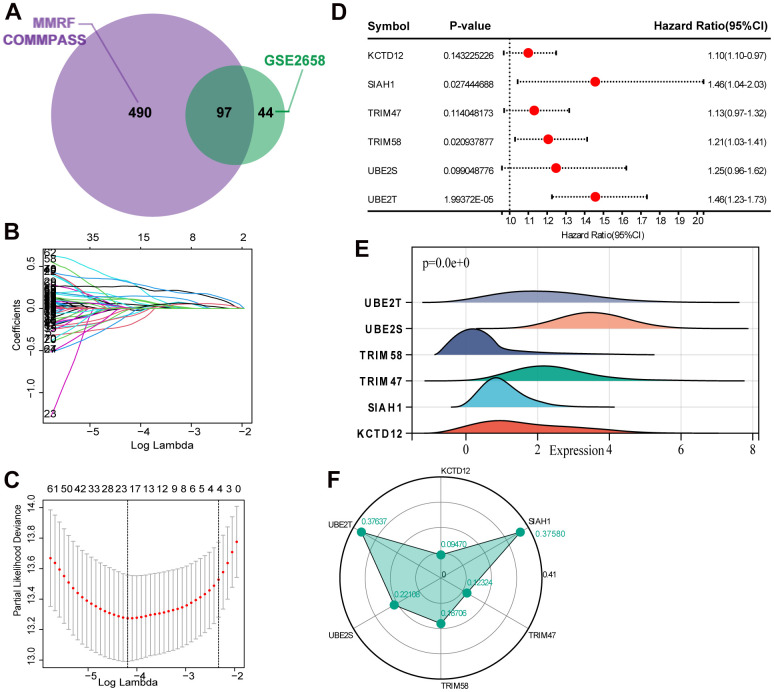
**Selection of robust biomarkers to establish a prognostic UPS gene signature.** (**A**) The 97 intersections of the OS related genes in MMRF-COMMPASS and GSE2658. (**B**) The LASSO coefficient profiles of the candidate OS-related UPS genes with nonzero coefficients. (**C**) A dotted vertical line represents the optimal value of the parameter (lambda) used in the LASSO model. (**D**) Multivariate Cox regression was used to establish the UPS gene signature, and six genes were finally selected as predictors of OS. (**E**) The mRNA levels of the 6 identified genes in training set (MMRF-COMMPASS). (**F**) Coefficient distribution of the gene signature.

We evaluated the expression profile of UPS genes associated with MM prognosis through a univariate Cox regression analysis. Based on the analysis of MMRF-COMMPASS data, we identified 490 UPS genes related to overall survival (OS) (Supplementary Table 2). In addition, we identified 141 UPS genes related to OS in GSE2658 (Supplementary Table 3). The 97 intersections of the overall survival related UPS genes were selected for the subsequent analysis (Figure 1A). Then, the Lasso-Cox proportional hazards regression and tenfold cross-validation were performed based on the 97 genes to generate the best gene model, and 20 candidate UPS genes were ultimately selected (Figure 1B, 1C). Furthermore, a multivariate Cox regression was performed to construct the UPS gene risk signature, and six genes were finally selected as predictors of OS (Figure 1D). Figure 1E shows the mRNA levels of the 6 identified genes. The weights assigned to each gene are shown in Figure 1F.

### Evaluation the reliability of the risk signature

**Figure 2:**
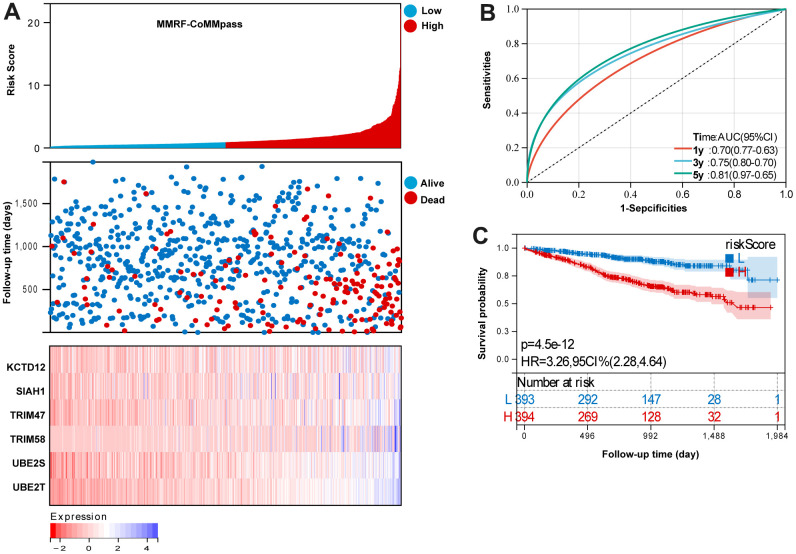
**Evaluation the reliability of the risk signature in the training set (MMRF-COMMPASS).** (**A**) summarizes the distribution of risk scores, the survival status of patients, and the expression of UPS genes in the training set. (**B**) A time-dependent ROC analysis for 1-, 3-, and 5-years OS prediction. (**C**) Survival analysis between low-risk and high-risk MM patients.

Based on the UPS signature was established to predict MM survival according to the formula: risk score = (KCTD12 × 0.09469841) + (SIAH1 × 0.375796476) + (TRIM47 × 0.123243103) + (TRIM58 × 0.187060727) + (UBE2S × 0.22107747) + (UBE2T × 0.37637129). In MMRF-COMMPASS training set, each patient’s risk score was calculated. The median was used to categorize the patients as either high-risk or low-risk. Figure 2A summarizes the distribution of risk scores, the survival status of patients, and the expression of UPS genes in the training set. A time-dependent ROC analysis showed the AUC value were 0.70, 0.75, and 0.81 for 1-, 3-, and 5-years OS (Figure 2B). Additionally, survival analysis demonstrated that low-risk MM patients have significantly longer OS as compared with high-risk MM patients (Figure 2C).

### Diagnostic value of UPS genes

**Figure 3:**
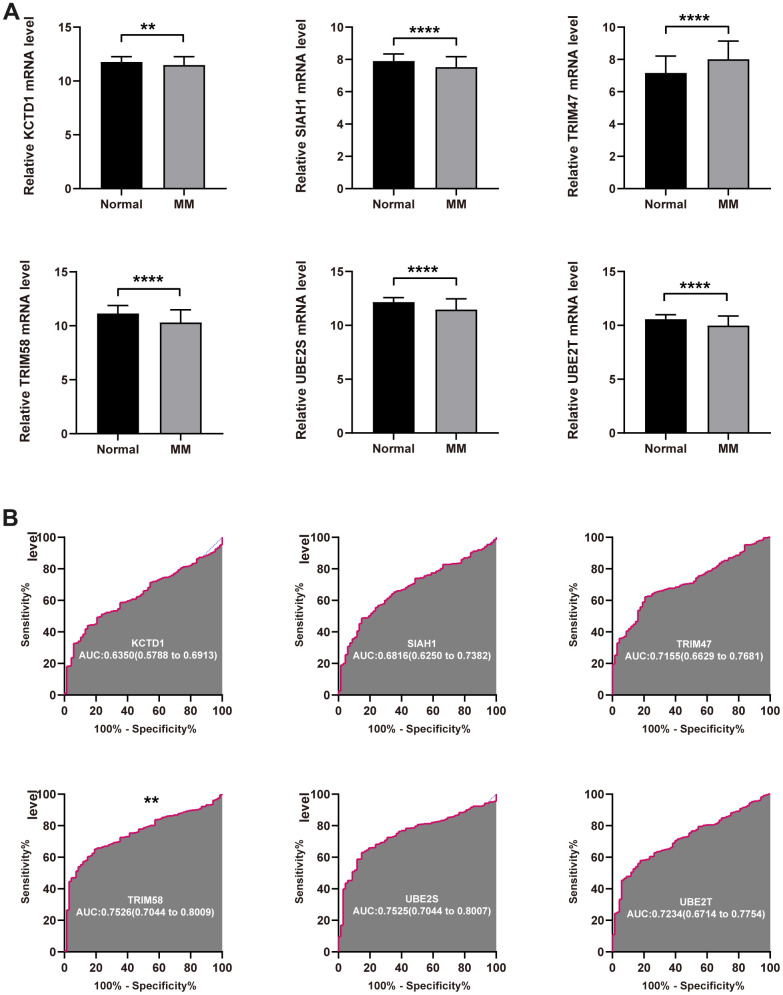
**Diagnostic value of the identified UPS genes for MM.** (**A**) The expression level of KCTD12, SIAH1, TRIM58, TRIM47, UBE2S, and UBE2T in tumor normal tissue. (**B**) ROC analysis showed the diagnostic ability of KCTD12, SIAH1, TRIM58, TRIM47, UBE2S, and UBE2T to distinguish MM from the normal samples (**P<0.01 and ****P<0.0001).

As a first step, we compared the UPS genes expression in normal and tumor tissues in GSE118985 dataset. It was found that the expression level of KCTD12, SIAH1, TRIM58, UBE2S, and UBE2T were significantly decreased in tumor tissue compared with the normal tissue, but TRIM47 was upregulated (Figure 3A). We further investigated the diagnostic effectiveness of the six identified UPS genes. As displayed in the ROC analysis, the diagnostic ability of each gene to distinguish MM from the normal samples shows a superior diagnostic efficiency (Figure 3B).

### GSVA

**Figure 4:**
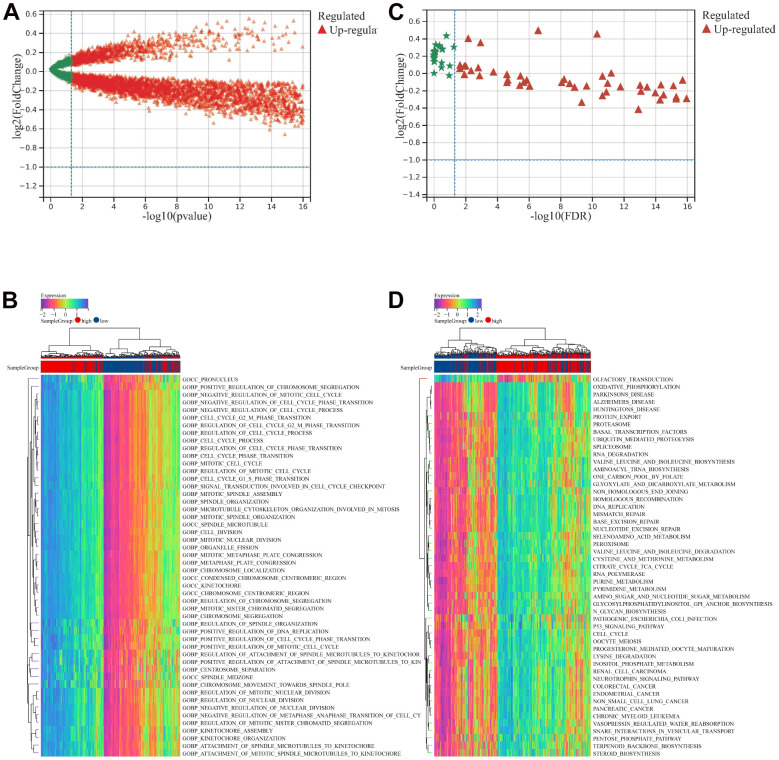
**GSVA analysis between low- and high- risk groups.** (**A**) Volcano map showed the enriched biological processes terms between low- and high- risk groups. (**B**) Top 50 enriched biological processes terms. (**C**) Volcano map showed the enriched KEGG pathways terms between low- and high- risk groups. (**D**) Top 50 enriched KEGG pathways terms.

We explored biological processes and KEGG pathways associated with risk signature using GSVA. As shown in Figure 4A, volcano map showed the enriched biological processes terms between low- and high- risk groups. Top five enriched biological processes terms were shown in Figure 4B*.* As shown in Figure 4C, volcano map showed the enriched KEGG pathways between low- and high- risk groups. Top five enriched KEGG pathways were shown in Figure 4D.

### Validation the UPS signature

Risk scores were calculated for each patient in the validation sets, including GSE2658, GSE136377, and GSE57317, and the median was used to categorize the patients as either high-risk or low-risk.

**Figure 5:**
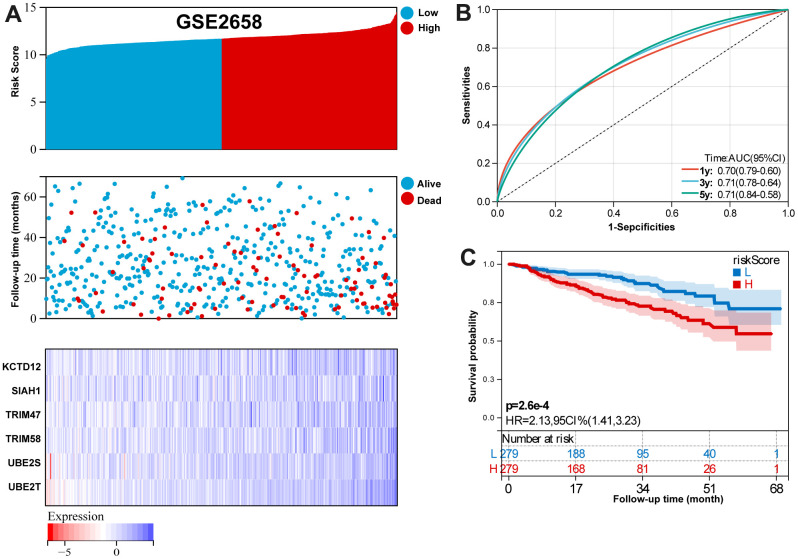
**Validation the reliability of the risk signature in the validation set (GSE2658).** (**A**) summarizes the distribution of risk scores, the survival status of patients, and the expression of UPS genes in the training set. (**B**) A time-dependent ROC analysis for 1-, 3-, and 5-years OS prediction. (**C**) Survival analysis between low-risk and high-risk MM patients.

For GSE2658 validation set, Figure 5A summarizes the distribution of risk scores, the survival status of patients, and the expression of UPS in the training set. A time- dependent ROC analysis showed the AUC value were 0.70, 0.71, and 0.71 for 1-, 3-, and 5-years OS (Figure 5B). Additionally, survival analysis demonstrated that low-risk MM patients have significantly longer OS as compared with high-risk MM patients (Figure 5C).

**Figure 6:**
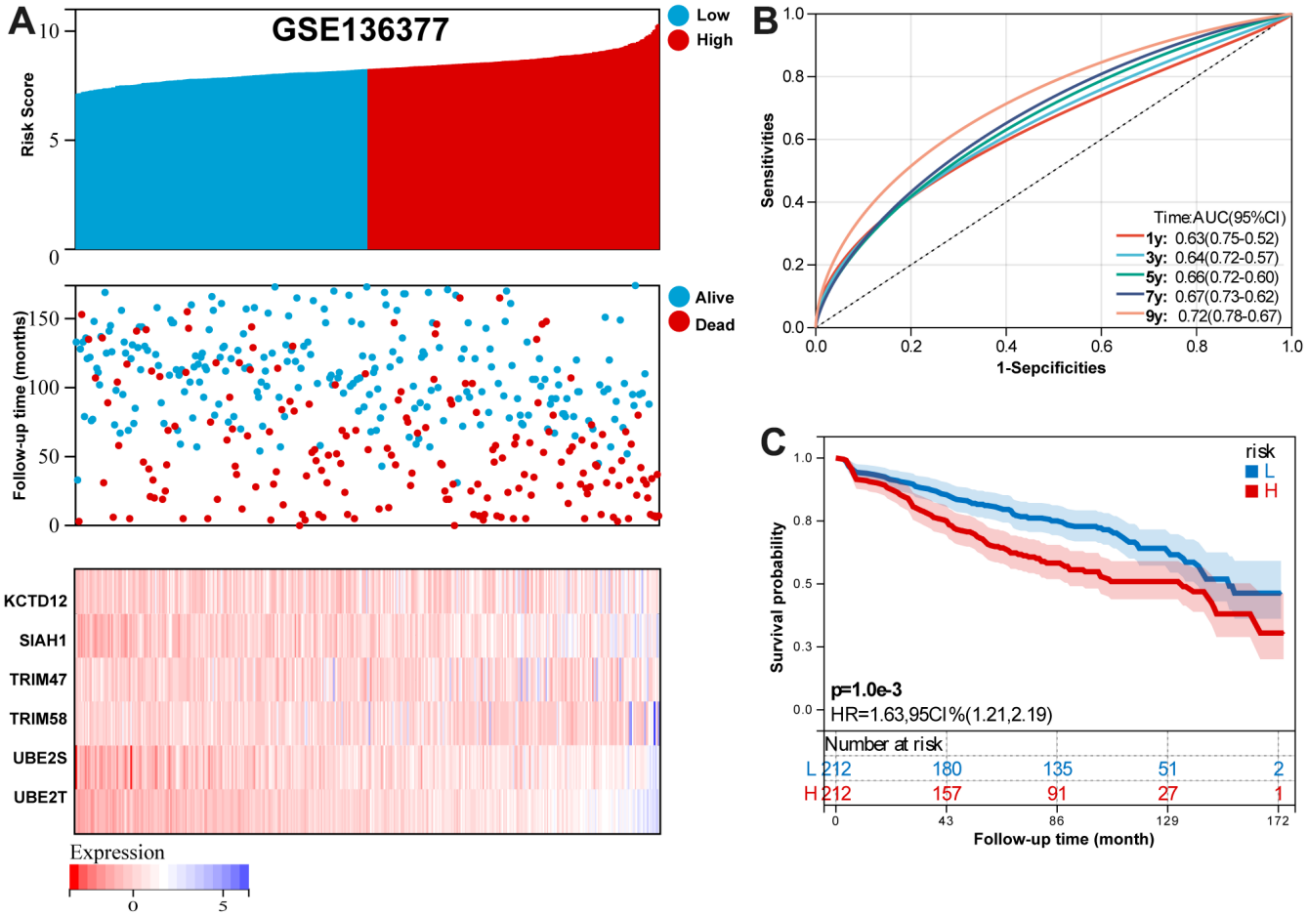
**Validation the reliability of the risk signature in the validation set (GSE136377).** (**A**) summarizes the distribution of risk scores, the survival status of patients, and the expression of UPS genes in the training set. (**B**) A time-dependent ROC analysis for 1-, 3-, 5-, 7-, and 9-years OS prediction. (**C**) Survival analysis between low-risk and high-risk MM patients.

For GSE136377 validation set, Figure 6A summarizes the distribution of risk scores, the survival status of patients, and the expression of RBP in the training set. A time-dependent ROC analysis showed the AUC value were 0.63, 0.64, 0.66, 0.67, and 0.72 for 1-, 3-, 5-, 7-, and 9-years OS (Figure 6B). Additionally, survival analysis demonstrated that low-risk MM patients have significantly longer OS as compared with high-risk MM patients (Figure 6C).

**Figure 7:**
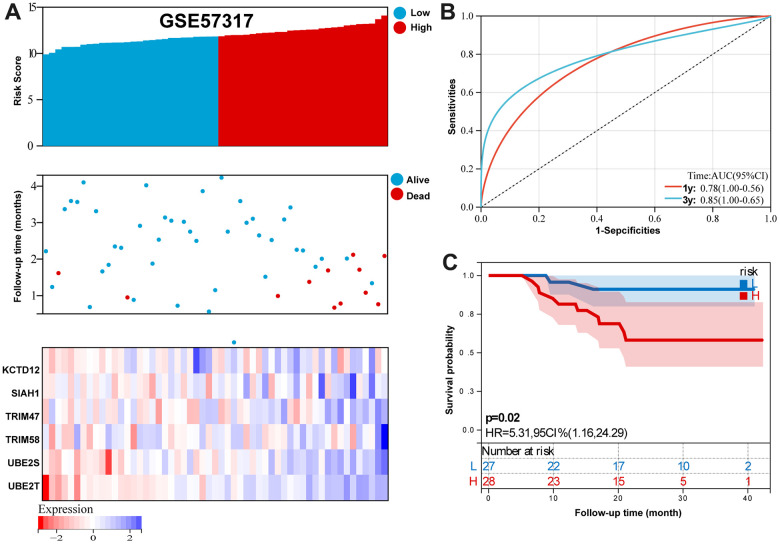
**Validation the reliability of the risk signature in the validation set (GSE57317).** (**A**) summarizes the distribution of risk scores, the survival status of patients, and the expression of UPS genes in the training set. (**B**) A time-dependent ROC analysis for 1- and 3-years OS prediction. (**C**) Survival analysis between low-risk and high-risk MM patients.

For GSE57317 validation set, Figure 7A summarizes the distribution of risk scores, the survival status of patients, and the expression of RBP in the training set. A time-dependent ROC analysis showed the AUC value were 0.78 and 0.85 for 1-, and 3-years OS (Figure 7B). Additionally, survival analysis demonstrated that low- risk MM patients have significantly longer OS as compared with high-risk MM patients (Figure 7C).

### Immune cell infiltration estimation

To further study immune characteristics in MM with a different immune risk score. We investigated the infiltrating immune cells by using the CIBERSORT algorithm. Supplementary Figure 1A illustrates the percentage of immune cells infiltrating the tumor. A comparison was made between high-risk and low-risk groups in terms of immune infiltration levels of a variety of immune infiltrating cells. The results showed High-risk participants had higher proportions of plasma cells, T cells CD8, T cells CD4 memory resting, NK cells activated, Dendritic cells activated, and Eosinophils (Supplementary Figure 1B).

### Independent prognostic factor

In order to identify independent risk factors for MM, a univariate and multivariate Cox regression analyses was performed as described above. The results indicated that age, stage, and risk score were the independent prognostic indicator in both MMRF-COMMPASS (Supplementary Figure 2A, 2B). Moreover, age, stage, and risk score also served as the independent prognostic indicators in GSE136377 (Supplementary Figure 2C, 2D).

### Construction of a prognostic nomogram

**Figure 8:**
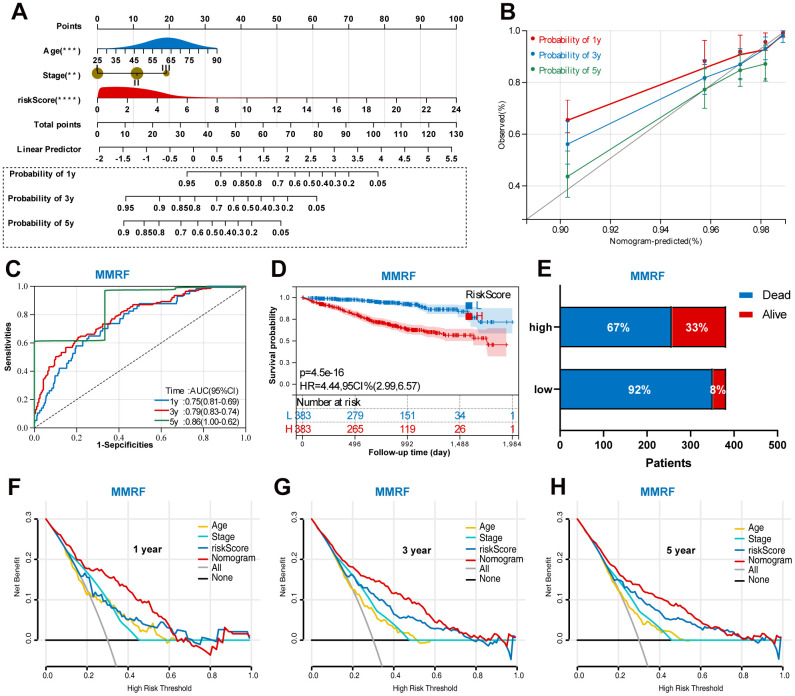
**Construction of a prognostic nomogram in the training set based on the independent risk factors.** (**A**) Nomogram based on the age, stage, and UPS signature. (**B**) Calibration plot of the nomogram for the prediction of OS. (**C**) A time-dependent ROC analysis for 1-, 3-, and 5-years OS prediction. (**D**) Survival analysis between low-risk and high-risk MM patients. (**E**) The relative proportion of alive and death cases between two groups. (**F**) DCA of the nomogram for the prediction of 1-year OS. (**G**) DCA of the nomogram for the prediction of 3-year OS. (**H**) DCA of the nomogram for the prediction of 5-year OS.

Based on the training set, we established a nomogram for accurate clinical prediction of MM survival, by independent prognostic factors, including age, stage, and risk score (Figure 8A). The C-index values were 0.76 in OS nomogram. The calibration plots based on the training set showed good agreement between predictions and observations (Figure 8B). Time-dependent ROC analysis showed the AUC value were 0.75, 0.79, and 0.86 for 1-, 3-, and 5-years OS (Figure 8C). Additionally, survival analysis demonstrated that low-risk MM patients have significantly longer OS as compared with high-risk MM patients (Figure 8D). The results also showed MM exhibited a greater mortality risk with an increasing risk score (Figure 8E). Further, a decision curve analysis (DCA) was conducted for age, stage, signature risk score, and nomogram, and showed that nomogram were clinically useful (Figure 8F–8H).

**Figure 9:**
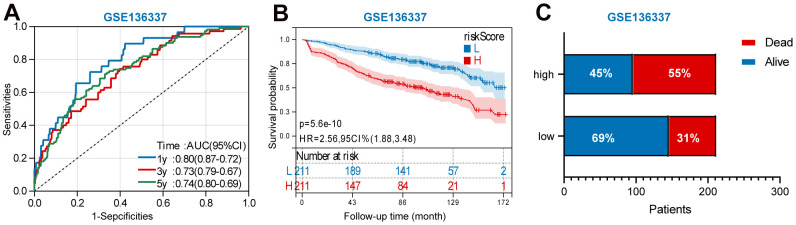
**Validation the prognostic nomogram in the validation set (GSE136377).** (**A**) A time-dependent ROC analysis for 1-, 3-, and 5-years OS prediction. (**B**) Survival analysis between low-risk and high-risk MM patients. (**C**)The relative proportion of alive and death cases between two groups.

In addition, we also performed a validation of the prognostic nomogram in the validation set (GSE136377). Time-dependent ROC analysis showed the AUC value were 0.80, 0.73, and 0.74 for 1-, 3-, and 5-years OS (Figure 9A). Survival analysis demonstrated that low-risk MM patients have significantly longer OS as compared with high-risk MM patients (Figure 9B). The results also showed MM exhibited a greater mortality risk with an increasing risk score (Figure 9C).

### Establishment of a web-based calculator

In order to make our findings more practical, we developed a web calculator (https://jiangyanxiamm.shinyapps.io/MMprognosis/) to predict the OS of MM according to the nomogram (Supplementary Figure 3A–3C). By drawing a perpendicular line from the total point axis to the outcome axis, we can estimate the odds of survival time.

## DISCUSSION

In the last decade, patients with MM have had significantly better outcomes and survival rates [21, 22]. As MM is complicated in its etiology and is difficult to predict in terms of prognosis. Improve the prognosis prediction of patients with MM through the development of new methods is also essential for making informed treatment decisions. In this study, we analyzed RNA-seq transcriptome profiles to develop a comprehensive UPS gene expression signature for MM prognosis. As far as we know, this is the first study to develop a UPS signature and nomogram for MM prognosis prediction.

In the current study, we developed an UPS-based signature of 6 genes, including KCTD12, SIAH1, TRIM58, TRIM47, UBE2S, and UBE2T. In the training set, the AUC value was 0.70, 0.75, and 0.81 for 1-, 3-, and 5-years OS prediction. Furthermore, we established a model based on independent prognosis factor. The multivariable model based on three features (age, stage, and risk score) showed promising predictive power in both training and validation sets. Based on our findings, this signature and model can be used to predict MM prognosis and assist in clinical decision-making.

Among the identified UPS genes, most of the genes were first identified as diagnostic and prognostic genes in MM. KCTD12 has been reported to be a prognostic biomarker of colorectal cancer and breast cancer [23, 24]. However, relevant research focused on MM remains limited. We found the downregulated KCTD12 expression and was associated worse outcome in MM. SIAH1, an E3 ubiquitin ligase, has been the topic of a range of investigations due to its varied functions both physiologically and pathologically, and its numerous new functions that have been identified [25]. There are a growing number of SIAH1 substrate proteins, which are mostly associated with fundamental cellular processes, including hypoxia responses, DNA damage responses, and cell division within cells [26–29]. There are reports that TRIM58 plays a role in a variety of cancers. Previous study reported that TRIM58 suppresses the tumor growth in tumor by inactivation of β-catenin signaling via ubiquitination [30]. There are, however, still questions regarding the expression level and functional role of TRIM58 in MM. A member of the TRIM family, TRIM47 plays an essential role in many cellular processes, including cell proliferation. It has been demonstrated that TRIM47 has E3 ligase activity, which is likely to play a role in tumor occurrence and prognosis [31]. Research shows tumor tissues express TRIM47 at a higher level than normal tissue [32], which is consistent with our findings. UBE2S is a ubiquitin-conjugating enzyme that is essential for the proper functioning of cellular processes [33]. The UBE2S gene is associated with a poor prognosis for cancers such as breast and gliomas, liver, and other malignant tumors [34]. UBE2T belongs to the E2 family of ubiquitin proteasomes. A variety of cellular functions are impacted by UBE2T, including DNA damage, genome instability, proliferation, and differentiation [35–37].

The UPS plays an important role in regulating immune cell function and response [38]. A comparison of immune cells types between low- and high-risk MM groups was performed here. The results showed High-risk participants had higher proportions of plasma cells, T cells CD8, T cells CD4 memory resting, NK cells activated, Dendritic cells activated, and Eosinophils. These results implied that these immune cells are associated with poor prognosis.

Nomograms are simple tools that create a visual representation of risk; they are extensively used in clinical settings to estimate risk. Clinical practitioners can use this tool to diagnose and estimate the prognosis of various patient groups since it has several key features. Our study used a nomogram to estimate the survival rate of MM patients based on their UPS risk score and other clinical features. Besides the classic nomogram, we developed a dynamic nomogram for estimating patient prognosis through web page operations. It may be more accurate to use a dynamic nomogram rather than previous nomograms that calculated an estimate.

A comprehensive analysis of UPS genes related to MM prognosis was conducted in our study. In this study, we developed a six-gene signature to predict patient outcomes with satisfactory prediction performance, but we encountered some limitations as well. First, since LUAD has a high degree of heterogeneity, some important clinical variables were not available from the public datasets. Therefore, greater number of clinical variables should therefore be included in future studies. Second, A deeper understanding of the mechanisms underlying the prognostic ability of the UPS genes in MM is required. Third, more independent MM cohorts should be used to validate the identified prognostic UPS genes.

In conclusion, our study developed and validated a prognostic model that includes the UPS signature as well as other clinical features in patients with MM, which performed well in predicting the survival of MM patients. It may be useful in determining treatment strategies and potential outcomes for MM using this model.

## Supplementary Material

Supplementary Figures

Supplementary Table 1

Supplementary Table 2

Supplementary Table 3
