# Supplementary material for: A nomogram for predicting prognosis of multiple myeloma patients based on a ubiquitin-proteasome gene signature
Source: Aging (Albany NY). 2022 Dec 18;14(24):9951–68. doi: 10.18632/aging.204432 (PMC9831738; doi:10.18632/aging.204432)
Supplement: Supplementary Figures [file aging-14-204432-s001.pdf]

SUPPLEMENTARY FIGURES

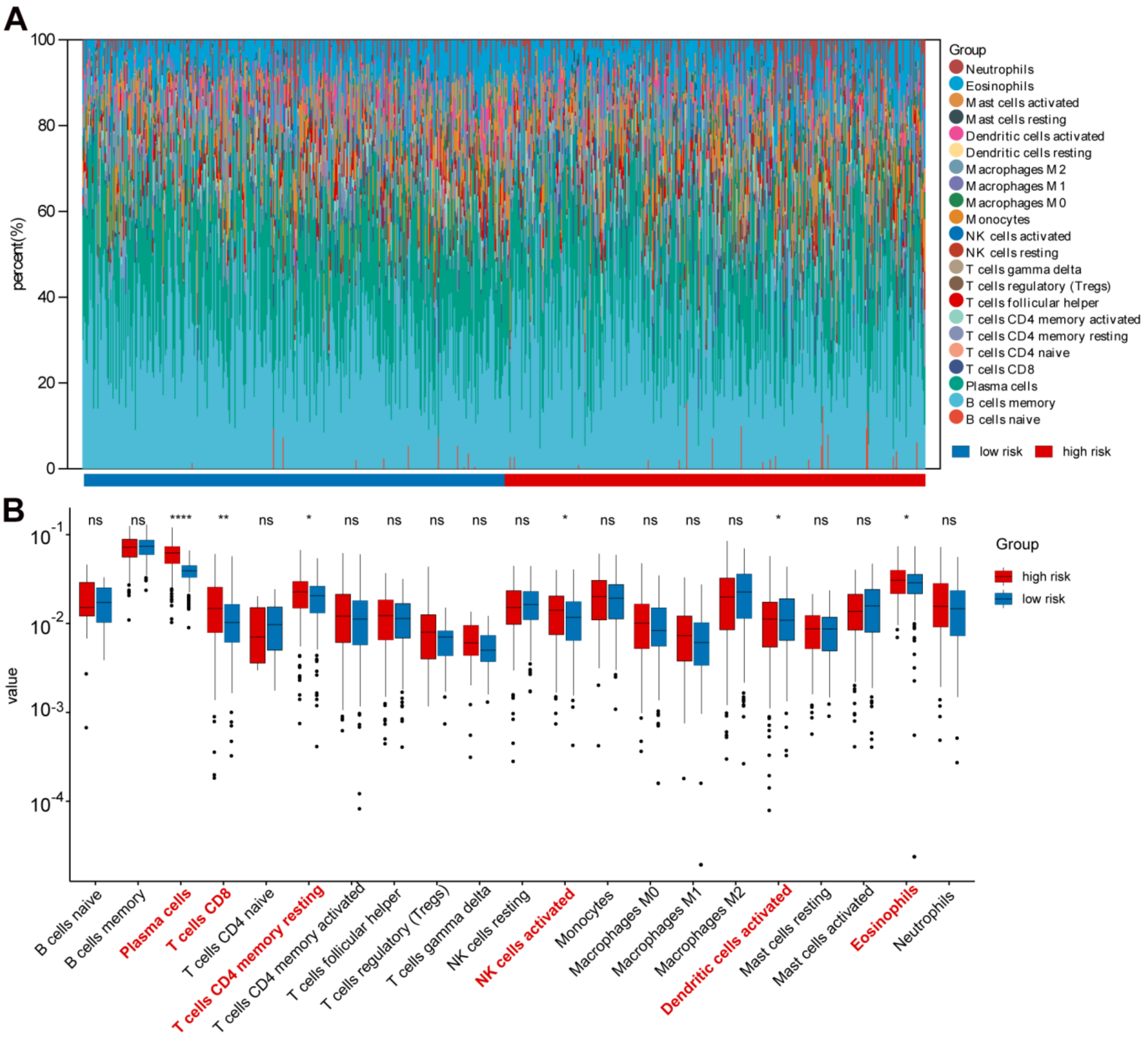

Supplementary Figure 1. Immune cell infiltration differences between low-risk groups and high-risk groups of MM in MMRF-COMMPASS. (**A**) Relative proportions of immune cells between different groups. (**B**) Vioplot visualization of significantly different proportions of immune cells between different groups (*<0.05, **<0.01, and ***<0.001).

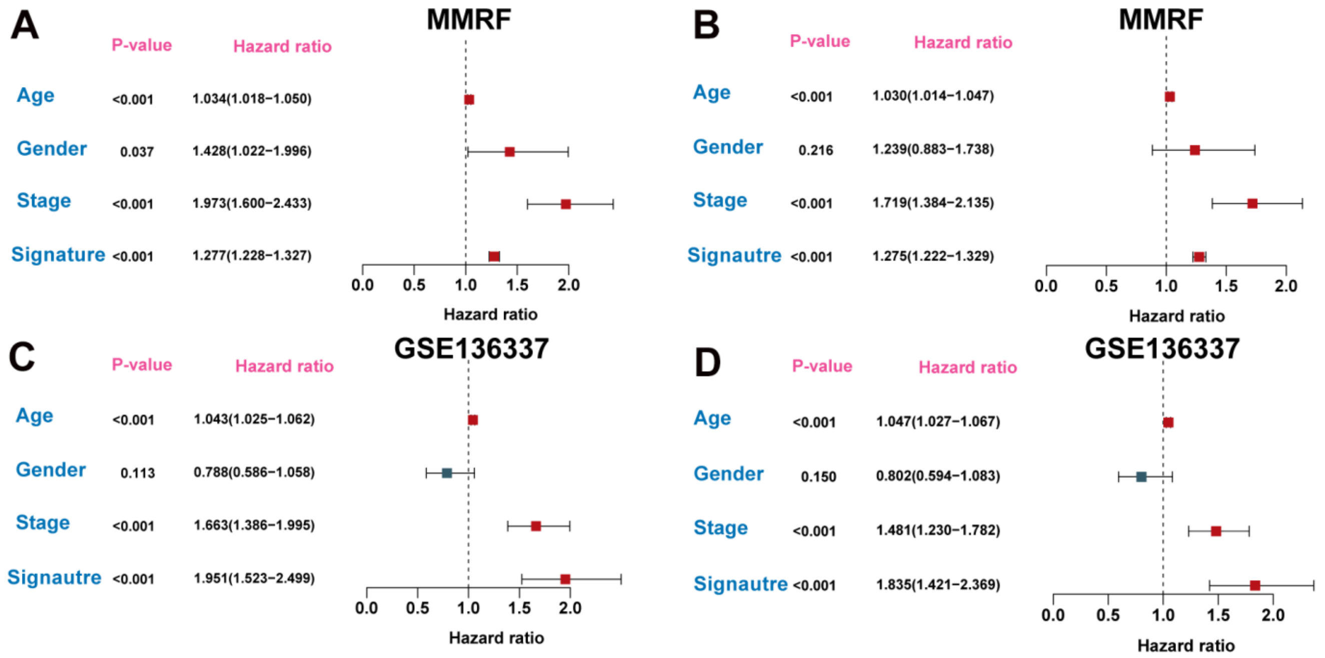

Supplementary Figure 2. Identification of independent risk factors for MM based on univariate and multivariate analysis. (**A**) Univariate analysis for MMRF. (**B**) Multivariate analysis for MMRF. (**C**) Univariate analysis for GSE136377. (**D**) Multivariate analysis for GSE136377.

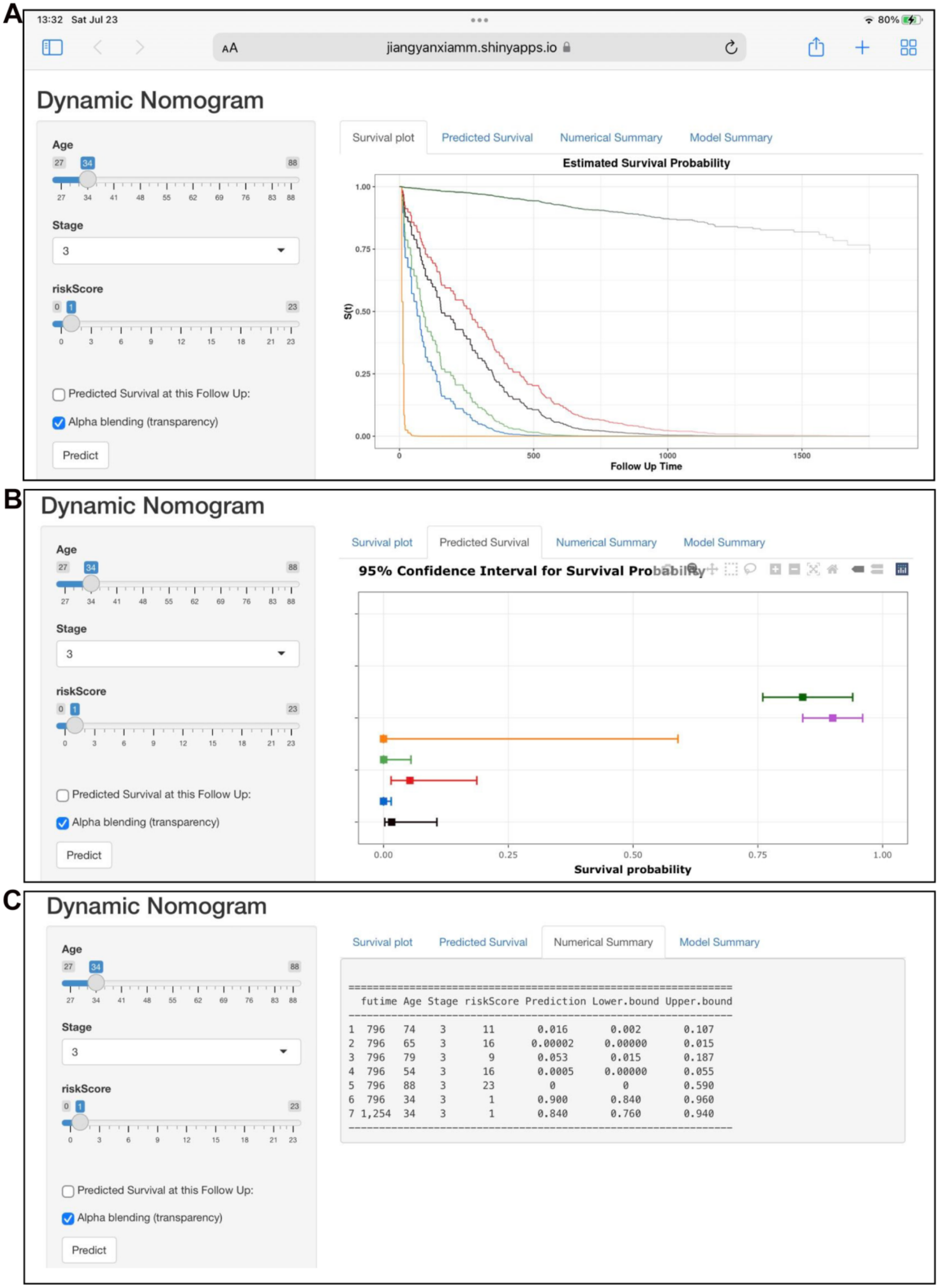

Supplementary Figure 3. Predicting OS of MM patients via a web-based dynamic nomogram (https://jiangyanxiamm.shinyapps.io/MMprognosis/). (**A**) Web survival rate calculator. (**B**) 95% confidence interval of the survival probability. (**C**) Numerical summary of different cases.
